# Effectiveness of Contrast-Enhanced Spectral Mammography Following Contrast-Enhanced Computed Tomography in Breast Cancer Diagnosis

**DOI:** 10.3390/diagnostics16071062

**Published:** 2026-04-01

**Authors:** Iksan Tasdelen, Ahmet Gunkan, Fatma Nur Soylu

**Affiliations:** 1 Department of General Surgery, Fatih Sultan Mehmet Training and Research Hospital, 34758 Istanbul, Turkey; 2 Division of Vascular and Interventional Radiology, Department of Radiology, University of Arizona, Tucson, AZ 85724, USA; gunkanahmet@gmail.com; 3 Department of Radiology, Radiologica Imaging and Diagnostic Center, 34846 Istanbul, Turkey; nursoylu@yahoo.com

**Keywords:** contrast-enhanced spectral mammography, contrast-enhanced CT, sequential imaging, breast cancer diagnosis, diagnostic performance

## Abstract

**Background:** Contrast-enhanced spectral mammography (CEM) provides functional information on tumor vascularity and is increasingly used for breast imaging, particularly as an alternative to breast MRI in selected clinical scenarios. During breast cancer staging, many patients undergo contrast-enhanced computed tomography (CT) and may subsequently receive a second dose of iodinated contrast for CEM, thus increasing contrast exposure, cost, and potential risk. This study aimed to evaluate the diagnostic effectiveness of CEM performed immediately after contrast-enhanced CT using the same contrast bolus (CT/CEM), in comparison with standard CEM requiring a separate contrast injection. **Methods:** The retrospective single-center study included 63 women with histopathologically confirmed breast cancer who underwent imaging between January of 2020 and December of 2024. Patients were divided into two groups: CT followed by CEM (CT/CEM, *n* = 29) and standard CEM alone (*n* = 34). **Results:** The CEM findings—including lesion visibility, enhancement characteristics, lesion conspicuity, background parenchymal enhancement (BPE), additional lesion detection, and tumor size—were assessed by two radiologists in consensus. The primary lesion was identified in all patients in both groups. No significant differences were found between groups in terms of lesion enhancement, conspicuity, BPE, or additional lesion detection (*p* > 0.05). Lesion conspicuity was higher in patients with low BPE, particularly in the CT/CEM group. **Conclusions:** CEM performed immediately after CT demonstrated diagnostic performance comparable to standard CEM, while eliminating the need for additional contrast administration.

## 1. Introduction

Breast cancer is the most frequently diagnosed malignancy among women worldwide and remains a major public health concern. Despite its high incidence, advances in strategies for its early detection, imaging, and multimodal treatment have significantly improved survival rates and enabled effective disease management [1].

Accurate staging plays a crucial role in determining prognosis, guiding treatment decisions, and planning surgical and systemic therapies. Imaging is central to this process, not only for evaluating the extent of the primary tumor but also for detecting multifocal, multicentric, and contralateral disease, as well as distant metastases [2]. Therefore, optimizing imaging strategies while maintaining diagnostic accuracy and patient safety has become an important focus in modern breast cancer care.

Among the advanced techniques developed for breast imaging, contrast-enhanced spectral mammography (CEM) has emerged as a promising modality that combines morphologic and functional information [3]. Utilizing iodinated contrast material and dual-energy acquisition, CEM highlights tumor-related neoangiogenesis, allowing for enhanced visualization of malignant lesions when compared with conventional mammography [4]. In particular, this functional component improves lesion conspicuity in dense breast tissue, where the sensitivity of standard mammography may be limited.

Several studies have demonstrated that the diagnostic performance of CEM in lesion detection and characterization approaches that of breast MRI while offering advantages such as a shorter examination time, wider availability, lower cost, and fewer contraindications [5,6]. As a result, CEM is increasingly being incorporated into clinical practice for problem-solving, preoperative assessment, and evaluation of disease extent in patients with newly diagnosed breast cancer. Recent studies have demonstrated the abovementioned advantages of CEM, particularly in women with dense breast tissue [7,8,9,10,11]. When correlated with histopathologic findings, CEM has shown an excellent capability to estimate tumor size and detect multifocal or multicentric disease, thereby guiding treatment planning and surgical decision-making.

However, CEM requires intravenous administration of iodinated contrast material, which carries the potential for contrast-related reactions and rare adverse events [12,13]. In the staging setting, many patients already undergo contrast-enhanced computed tomography (CT) to evaluate distant metastases, meaning that they may receive iodinated contrast media more than once within a short period of time [14]. Consequently, this raises concerns regarding cumulative contrast dose, cost, workflow efficiency, and the potential risk of contrast-related adverse events in clinical practice [4,5].

A practical alternative approach is to perform CEM immediately after contrast-enhanced CT using the same contrast bolus, thereby eliminating the need for an additional contrast injection. Although a limited number of preliminary studies have explored this strategy, data regarding the optimal timing between CT and CEM acquisition remain scarce and associated strategies have not yet been standardized.

Therefore, this study aimed to evaluate the diagnostic effectiveness of CEM performed immediately after contrast-enhanced CT (CT/CEM), compared with standard CEM performed with a separate contrast injection. We hypothesized that sequential CT/CEM imaging would provide comparable lesion enhancement, lesion conspicuity, and background parenchymal enhancement (BPE), while enabling the use of a single-contrast bolus.

## 2. Materials and Methods

### 2.1. Study Design and Population

This retrospective, single-center study was conducted at our institution between January of 2020 and December of 2024. The study protocol was approved by the Institutional Ethics Committee of Fatih Sultan Mehmet Training and Research Hospital (Istanbul, Turkey) and was conducted in accordance with the principles of the Declaration of Helsinki. Given the retrospective design and the use of anonymized data, the requirement for informed consent was waived; however, written informed consent for publication of the radiological images presented in the figures was obtained from the respective patients.

The study population comprised 63 female patients with histopathologically confirmed breast cancer. Only patients with biopsy-proven malignancies were included, as the primary aim was to evaluate the diagnostic performance of CEM immediately after CT and the impact of contrast timing on lesion enhancement and conspicuity.

Patients were excluded if they had previously undergone contrast-enhanced spectral mammography (CEM) or contrast-enhanced thoracoabdominal computed tomography (CT) for breast cancer staging; had undergone positron emission tomography/computed tomography (PET/CT); had elevated serum creatinine levels; were pregnant or suspected of being pregnant; or had a known allergy to iodinated contrast material.

Patients were divided into two groups: those who underwent CEM immediately following contrast-enhanced CT (CT/CEM group; *n* = 29) and those who underwent standard CEM alone (CEM group; *n* = 34). In both groups, CT examinations were performed at the time of initial diagnosis for staging purposes prior to treatment, based on similar clinical staging indications. Group allocation was determined according to the workflow and timing of contrast administration. Demographic characteristics, histopathologic subtypes, and imaging parameters were recorded for all patients.

### 2.2. Imaging Technique and Interpretation

#### 2.2.1. CT Technique

All CT examinations were performed using a 16-channel multidetector row CT scanner (Somatom Emotion; Siemens Healthineers, Erlangen, Germany). Intravenous access was established with a 20-gauge catheter (BD Venflon™, Becton Dickinson, Helsingborg, Sweden), and low-osmolar iodinated contrast material (iopamidol; Iopamiron 300 mg I/mL, Bracco Imaging S.p.A., Milan, Italy) was administered at a flow rate of 2.5 mL/s, for a total dose of 1.5 mL/kg body weight (with a maximum contrast volume of 120 cc).

The start of contrast injection was defined as T0 in both groups. Contrast-enhanced imaging commenced approximately 90–120 s after contrast injection, where the scanning range included the thorax and upper abdomen. The main acquisition parameters were as follows: a tube voltage of 120 kVp, a tube current of 150–180 mAs, 16 × 1.5 mm collimation, a rotation time of 0.5 s, and a normalized pitch of 1 (Table 1).

#### 2.2.2. CEM Technique

In the CT/CEM group, patients were transferred immediately to the mammography suite located adjacent to the CT room and underwent CEM without additional contrast administration. In this group, the interval between contrast injection and the first CEM exposure ranged from 3 min 30 s to 6 min (mean ± SD, 4.20 ± 0.53 min).

In the CEM-only group, intravenous contrast material was administered in the mammography suite using the same injection protocol as for the CT/CEM group (iopamidol 300 mg I/mL, 1.5 mL/kg body weight at 2.5 mL/s, with a limit on maximum contrast volume of 120 cc). In this group, the interval between contrast injection and the first CEM exposure ranged from 2 min to 2 min 20 s (mean ± SD, 2.10 ± 0.43 min). Detailed contrast administration and timing parameters for both the CT/CEM and CEM-only groups—including the temporal reference point, injection characteristics, transfer time, and acquisition timing—are summarized in Table 2.

All CEM examinations were performed using a Senographe DS system (GE Healthcare, Chalfont St. Giles, UK), a full-field digital mammography unit equipped for dual-energy acquisition. Two minutes after contrast administration, low- and high-energy images were obtained using dedicated software. Low- and high-energy images were acquired at 26–31 kVp and 45–49 kVp, respectively. The tube voltage and tube current (kVp and mAs) were automatically adjusted according to breast thickness.

The imaging protocol for both groups began with a craniocaudal (CC) view of the affected breast, followed by a CC view of the contralateral breast and mediolateral oblique (MLO) views of both breasts. In both groups, all CEM projections were completed within 10 min from the start of contrast injection (T0), ensuring that image acquisition occurred within the early post-contrast phase. Subtracted images were generated automatically using software that removed background parenchymal structures and non-contrast-enhancing lesions. The imaging parameters used for the CEM examination are summarized in Table 3.

#### 2.2.3. Image Interpretation

All CEM images were anonymized and evaluated in consensus by two radiologists using a dedicated mammography workstation (Seno Iris; GE Healthcare, Buc, France). Radiologist A had 20 years of experience in breast imaging, while Radiologist B had 1 year of experience. Image interpretation was performed in four separate reading sessions, with cases presented in a randomized order.

Both radiologists were blinded to group allocation (CT/CEM vs. CEM-only) and all clinical and histopathologic information, except for the knowledge that each patient had biopsy-proven breast cancer.

**Figure 1:**
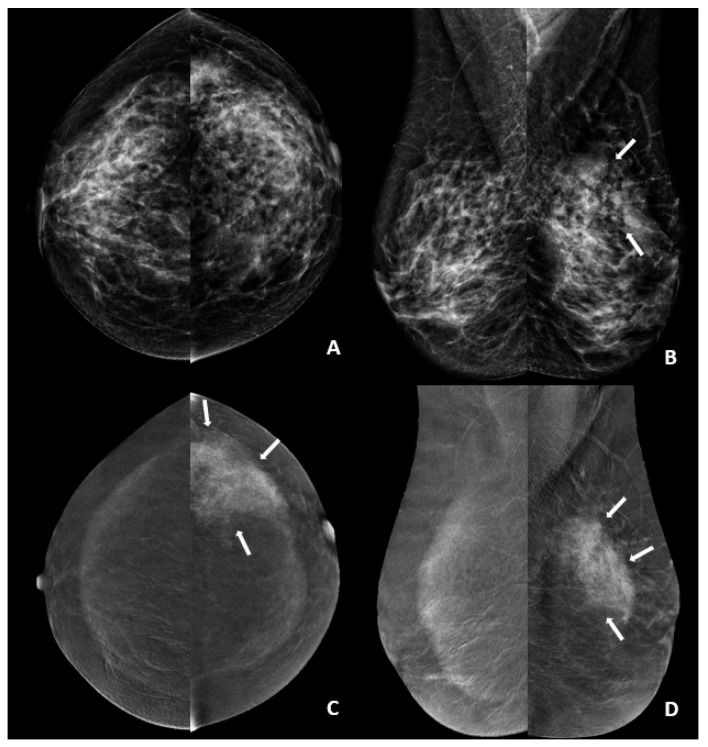
CEM images after CT examination of a 44-year-old female patient with left breast IDC diagnosis. (**A**,**B**) Low-energy CC and MLO images show an asymmetry and parenchymal distortion area (arrows) in the upper outer quadrant of the left breast. (**C**,**D**) Subtracted images show non-mass pathological contrast uptake in this area (arrows), with a maximum size of 75 mm measured on subtraction images. The scores for this patient were as follows: lesion enhancement, 4; conspicuity of the primary lesion, 4; and background parenchymal enhancement, 1.

For each case, three imaging parameters were evaluated in consensus: (1) degree of contrast enhancement of the primary tumor, (2) conspicuity of the primary lesion, and (3) background parenchymal enhancement (BPE). Lesion enhancement and conspicuity were additionally assessed in relation to the degree of BPE in both groups (Figure 1).

Each parameter was rated using a 4-point visual scale. Background parenchymal enhancement (BPE) was categorized according to the BI-RADS terminology as minimal, mild, moderate, or marked. Lesion enhancement intensity and lesion conspicuity were graded using a 4-point visual scale reflecting increasing degrees of contrast uptake and lesion visibility.

Lesion enhancement intensity was scored as follows:

1. Barely perceptible enhancement.
2. Definite but low-intensity enhancement.
3. Clearly visible enhancement.
4. Intense enhancement.

Lesion conspicuity was scored as follows:

1. Lesion difficult to distinguish from surrounding tissue.
2. Lesion identifiable but not clearly delineated.
3. Lesion clearly distinguishable from surrounding tissue.
4. Lesion readily identifiable with strong visual contrast from adjacent parenchyma.

The same scoring framework was applied uniformly across all cases in both groups.

For secondary analysis, these parameters were dichotomized as present or absent.

**Figure 2:**
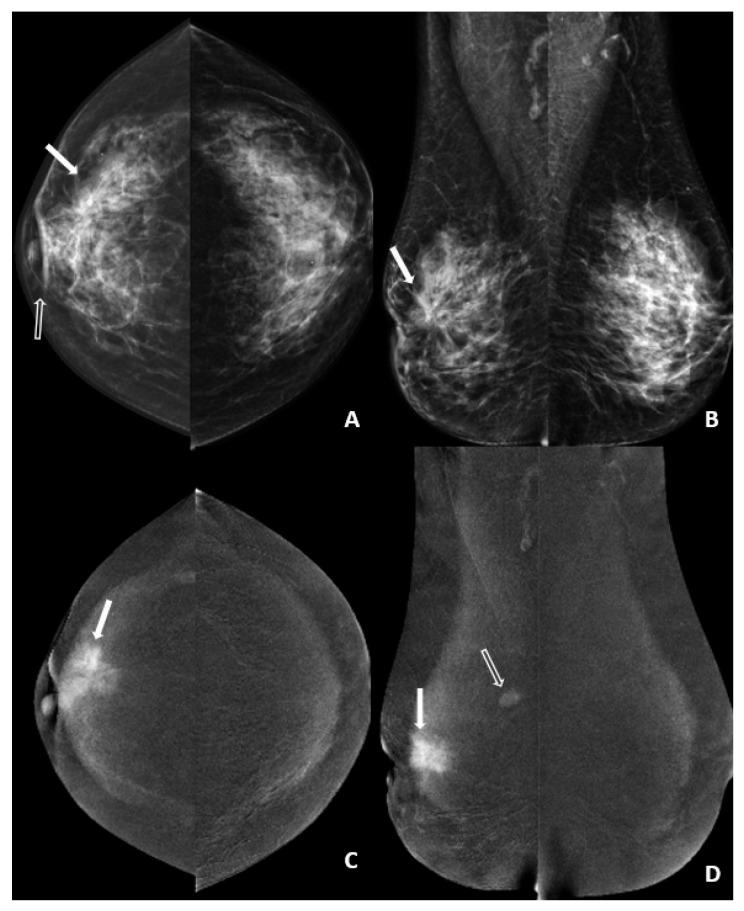
Standard CEM images after CT examination of a 49-year-old female patient with right breast IDC diagnosis. (**A**,**B**) Low-energy CC and MLO images show parenchymal distortion area (arrow) in the upper outer quadrant of the right breast with skin and nipple contraction (open arrow). (**C**,**D**) Subtracted images show a contrast-enhanced mass with a maximum diameter of 42 mm and an additional enhancing focus in the same quadrant (open arrow). The scores for this patient were as follows: lesion enhancement, 4; conspicuity of the primary lesion, 4; and background parenchymal enhancement, 1.

Tumor size estimation was based on the maximum linear extent of enhancement observed in recombined CEM images. Measurements were obtained from the projection (craniocaudal or mediolateral oblique) demonstrating the greatest enhancing tumor extent. When spiculated morphology or non-mass lesions (e.g., architectural distortion) was present, the measurement incorporated the entire enhancing abnormality, including radiating extensions or the distorted breast parenchyma. Pathological measurements were obtained from routine histopathological evaluation of the surgical specimen. Only the invasive component size was considered for correlation with imaging findings. The presence of additional enhancing foci and axillary lymph node involvement was recorded. These imaging findings were subsequently compared with the histopathologic results (Figure 2).

### 2.3. Statistical Analysis

All statistical analyses were performed using IBM SPSS Statistics for Windows, version 22 (IBM Corp., Armonk, NY, USA). The Kolmogorov–Smirnov and Shapiro–Wilk tests were applied to assess the normality of data distributions. Descriptive statistics are expressed as mean ± standard deviation or frequency, as appropriate.

Comparisons of quantitative variables between groups were conducted using Student’s *t*-test. Qualitative variables were compared using Fisher’s exact test or the chi-square test with Yates continuity correction, as appropriate.

All *p*-values are two-sided, with a value less than 0.05 considered statistically significant.

## 3. Results

A total of 63 patients were included in the study (age range, 31–79 years; mean age, 55.46 ± 11.75 years). These patients were divided into two groups, according to the imaging protocol: a CT/CEM group, in which contrast-enhanced spectral mammography was performed immediately after contrast-enhanced CT using the same contrast bolus, and a CEM group, in which CEM was performed with a separate contrast injection. The distribution of histopathologic breast cancer subtypes in each group is presented in Table 4.

A comparison of the groups in terms of age and maximum diameter of the primary lesion is presented in Table 5. There was no statistically significant difference between the two groups with respect to age (*p* = 0.876), indicating that they were comparable in terms of demographic characteristics. This similarity suggests that any potential age-related bias in the comparison of imaging findings was likely limited.

In contrast, the mean diameter of the primary lesion was significantly larger in the CT/CEM group than in the CEM-only group (42.82 ± 23.03 mm vs. 29.15 ± 15.95 mm; *p* = 0.008). This finding suggests that patients in the CT/CEM group may have had larger tumors, and this difference was taken into consideration during the interpretation of the imaging findings.

No statistically significant differences were observed between the groups regarding the degree of primary lesion enhancement, background parenchymal enhancement (BPE), or lesion conspicuity (*p* > 0.05) (Table 6). In the CT/CEM group, all primary lesions demonstrated enhancement (100%), and the majority of lesions in the CEM group also showed enhancement (94.1%). Similarly, the distribution of BPE was comparable between the groups, with marked parenchymal enhancement observed in 37.9% and 41.2% of cases in the CT/CEM and CEM groups, respectively.

Lesion conspicuity was also similar between the groups, with the majority of lesions considered conspicuous in both the CT/CEM and CEM groups (86.2% vs. 91.2%, respectively). These findings indicate that CEM performed after contrast-enhanced CT provides imaging quality and lesion enhancement characteristics comparable to standard CEM.

According to the histopathologic findings, both groups demonstrated high sensitivity and specificity for the detection of lymph node involvement and additional enhancing foci, with no statistically significant differences between groups (*p* > 0.05).

Lesion enhancement and lesion conspicuity were evaluated in the CT/CEM group, according to the level of background parenchymal enhancement (BPE) (Table 7). Lesion enhancement was present in all cases and was rated as good, regardless of the degree of BPE. In contrast, lesion conspicuity varied according to the BPE level. All lesions in patients with low BPE were considered conspicuous (100%), whereas only 63.6% of lesions in patients with high BPE were rated as conspicuous. This difference was statistically significant (*p* = 0.014).

These findings suggest that increased background parenchymal enhancement may negatively affect lesion conspicuity.

In the CEM-only group, lesion enhancement and lesion conspicuity were also evaluated according to the level of background parenchymal enhancement (BPE) (Table 8). Lesion enhancement was present in 90% of patients with low BPE and in all patients (100%) with high BPE, with no statistically significant difference between the subgroups (*p* = 0.501).

Furthermore, lesion conspicuity was 100% in cases with low BPE and 78.6% in those with high BPE; however, this difference did not reach statistical significance (*p* = 0.061). These findings suggest that increased background parenchymal enhancement may tend to reduce lesion conspicuity in the CEM-only group, although this effect was not statistically significant.

The detection of additional lesions was observed at similar rates in both groups (Table 9), namely, with an incidence of 24.1% in the CT/CEM group and 14.7% in the CEM-only group, with no statistically significant difference between the groups (*p* > 0.05). Regarding the degree of enhancement of additional lesions, all additional lesions in the CT/CEM group demonstrated enhancement (100%), while enhancement was observed in 60% of additional lesions in the CEM-only group; however, this difference was not statistically significant (*p* > 0.05).

In terms of conspicuity, all additional lesions were considered conspicuous in both groups (100%). The rates of lymph node involvement were also similar between the groups, being detected in 44.8% of patients in the CT/CEM group and 44.1% in the CEM-only group, again showing no statistically significant difference (*p* > 0.05).

These findings indicate that CEM performed after contrast-enhanced CT provides diagnostic performance comparable to standard CEM for the detection of additional lesions and lymph node involvement.

To further characterize the precision of the diagnostic performance estimates, confidence intervals were calculated for sensitivity and specificity. The results are presented in Table 10.

For lymph node detection, the sensitivity of CEM in the CT/CEM group was 71.4% (95% CI: 41.9–91.6) and the specificity was 60.0% (95% CI: 32.3–83.7); meanwhile, in the CEM-only group, the sensitivity was 77.8% (95% CI: 52.4–93.6) and the specificity was 68.8% (95% CI: 41.3–89.0). These results provide additional information regarding the precision of the estimated diagnostic performance measures.

To assess the potential influence of confounding variables, a multivariate logistic regression analysis was performed with lesion detectability as the dependent variable and group status (CT/CEM vs. CEM-only) and primary tumor size as independent variables. The analysis revealed that group status did not have a statistically significant effect on lesion detectability (OR = 2.923, 95% CI: 0.527–16.211, *p* = 0.220). Similarly, primary tumor size was not significantly associated with lesion detectability (OR = 1.052, 95% CI: 0.989–1.119, *p* = 0.107). Model calibration was evaluated using the Hosmer–Lemeshow goodness-of-fit test, which indicated adequate model fit (χ^2^ = 10.173, *p* = 0.253).

## 4. Discussion

The present study demonstrated that contrast-enhanced spectral mammography (CEM) performed immediately after contrast-enhanced CT using the same intravenous contrast bolus provides diagnostic performance comparable to that of standard CEM, typically requiring an additional contrast injection. These findings further support the growing body of evidence indicating that CEM is a highly reliable functional breast imaging modality, particularly for the assessment of tumor extent and preoperative staging [3,7,8,9,15,16]. Although the study was retrospective and non-randomized, both groups shared identical clinical staging indications, and allocation was determined according to the workflow and timing of contrast administration, rather than disease severity.

Previous comparative studies have demonstrated that CEM allows for the detection of index lesions and estimation of tumor size with accuracy comparable to that of MRI, while maintaining superiority over conventional mammography in women with dense breast tissue [15]. Meta-analyses have further demonstrated that CEM provides high pooled sensitivity and specificity across a range of clinical settings, including preoperative staging and screening [7,16]. In addition, systematic reviews have indicated that CEM may serve as a viable alternative to MRI in many patients, particularly when cost, limited availability, or contraindications restrict the use of MRI [8,9]. Collectively, these findings support the effectiveness of CEM as a robust diagnostic tool in breast cancer care.

Our study is consistent with this growing body of evidence and extends it by demonstrating that sequential CT/CEM imaging does not compromise lesion visibility, enhancement, or conspicuity. These findings are in line with prior investigations, including the study by Okada et al., who reported no significant differences in lesion visibility when CEM was performed immediately after CT, even across variable injection-to-imaging intervals [10]. Similarly, other studies evaluating CEM performance using extended or delayed acquisition protocols have shown that contrast enhancement remains sufficient for reliable diagnostic interpretation over a broad range of timing intervals [9,10,11]. Collectively, these results suggest that a rigid timing window may not be required to achieve optimal CEM performance. It should be noted that a statistically significant difference in primary tumor diameter was observed between the groups in this study, with larger lesions in the CT/CEM cohort. While multivariate analysis did not demonstrate a significant independent effect of tumor size on lesion detectability, this baseline imbalance may still represent a potential source of bias. Larger tumors are generally easier to detect and may exhibit more prominent enhancement, which could influence subjective assessments of lesion conspicuity. Therefore, although our adjusted analyses suggest comparable diagnostic performance, the findings should be interpreted with caution.

A notable finding of our study is the consistent relationship between background parenchymal enhancement (BPE) and lesion conspicuity; specifically, lesion conspicuity decreased in cases with high BPE, reaching statistical significance in the CT/CEM group. The influence of BPE on diagnostic confidence has been well described, with increased enhancement of normal parenchyma potentially reducing the contrast-to-noise ratio and obscuring subtle lesions [17,18]. Hormonal factors—including menopausal status, estrogen levels, ovarian function, lactational physiology, and anti-estrogen therapy—are known contributors to variations in BPE [19,20]. Although lesion conspicuity was significantly affected by BPE in the CT/CEM group, this likely represents a physiological effect rather than a statistical artifact. The timing of CEM immediately after CT may coincide with early post-contrast enhancement of background parenchyma, transiently reducing lesion-to-background contrast. Prior studies have shown that contrast uptake remains adequate for reliable interpretation for up to 8–10 min across dual-energy acquisitions [19,20,21,22,23,24]. While the observed significance may partly reflect the relatively small sample size—particularly in the subgroup analyses—this finding should be interpreted cautiously and in line with prior studies [17,18,19,20,21,22,23,24]. Our findings support this concept and indicate that, although BPE affects lesion conspicuity, sequential CT/CEM remains diagnostically robust within the first several minutes following contrast injection.

The practical advantages of sequential CT/CEM imaging are clinically meaningful. By avoiding a second contrast injection, this approach may reduce the overall iodine burden and has the potential to lower the risk of contrast-related adverse events, as suggested in previous studies [22,23,24]. From a workflow perspective, sequential imaging shortens the total examination time, eliminates the need for repeat venipuncture, and reduces procedural costs. Collectively, these factors can enhance patient comfort and improve departmental efficiency. Moreover, when CT and mammography units are physically adjacent (as in our institution), patient transfer times are minimal, facilitating seamless implementation of this protocol within routine staging workflows. This streamlined approach may be particularly advantageous in high-volume breast imaging centers.

Although the sequential CT/CEM approach may have potential implications for contrast utilization and examination workflow, these aspects were not directly evaluated in the present study and therefore should be interpreted cautiously. Prospective investigations specifically designed to assess safety outcomes, workflow metrics, and cost-effectiveness would be necessary to confirm these potential advantages.

Additionally, the diagnostic performance of CEM for detecting additional enhancing foci and axillary lymph node involvement in our study was consistent with previously reported sensitivities and specificities [25]. These findings indicate that sequential CT/CEM imaging preserves diagnostic reliability for the detection of clinically relevant secondary lesions, which is critical for accurate staging and informed treatment planning.

Despite these findings, several limitations should be considered when interpreting the results. First, the relatively small sample cohort may limit the study’s statistical power, particularly regarding subgroup analyses such as the BPE-related comparisons and additional lesion detection. In addition to the small sample size, the retrospective study design may limit the generalizability of our findings, as is the case for many early-stage CEM investigations [3,7,8,15,16]. Second, the inclusion of only biopsy-proven malignancies precludes an evaluation of specificity and limits the assessment of diagnostic performance in a real-world clinical setting, where differentiation between benign and malignant lesions is required. Third, the relatively small number of invasive lobular carcinoma cases limited the ability to perform a meaningful subgroup analysis according to histologic subtype. Fourth, the feasibility of this protocol depends on the physical proximity of CT and mammography units, which may pose a logistical constraint and limit applicability across imaging centers. Fifth, image interpretation was performed in consensus by two radiologists, rather than independently. Therefore, inter-observer agreement was not assessed, which limits evaluation of reproducibility, particularly considering that a subjective visual scoring system was used. Although independent evaluation with kappa statistics could provide additional information regarding reproducibility, the primary objective of the study was to compare lesion enhancement characteristics between groups under different contrast timing conditions, rather than to evaluate observer variability. Consensus reading was selected to minimize interpretative discrepancies and reflect routine clinical practice. Finally, enhancement assessment was based on qualitative visual scoring rather than quantitative measurements such as signal-to-noise ratio or contrast-to-noise ratio, which may have provided a more objective comparison between protocols. Future prospective studies incorporating independent readings and objective quantitative image analyses may further improve the assessment of reproducibility, thus providing a more comprehensive comparison between protocols.

Our findings indicate that CEM performed immediately after contrast-enhanced CT provides diagnostic performance comparable to that of standard CEM while eliminating the need for an additional contrast injection. This sequential approach preserved lesion visibility and conspicuity with respect to the evaluated parameters. Although the use of a single-contrast bolus may have implications for contrast utilization, cost, and workflow efficiency—with these potential advantages having been suggested in previous studies evaluating CEM implementation [26]—these aspects were not directly assessed in the present study and should therefore be interpreted with caution.

Given the robust body of evidence supporting CEM as a reliable alternative to MRI for functional breast imaging [11], the sequential CT/CEM protocol may represent a feasible addition to breast cancer staging workflows—particularly in institutions with integrated imaging environments [27]—although further prospective validation is required for confirmation.

## 5. Conclusions

In conclusion, CEM performed immediately after contrast-enhanced CT appears to provide diagnostic performance comparable to that of standard CEM while enabling the use of a single-contrast bolus. While this sequential approach may offer practical advantages, these findings should be interpreted in the context of the study’s limitations. Further prospective, larger-scale studies are required to validate the presented results and define the role of this approach within routine breast imaging workflows.

## Figures and Tables

**Table 1:** Imaging parameters for CT examination.

Detector rows	16
Tube voltage (kVp)	120
Tube current (mAs)	150–180
Rotation time (s)	0.5
Collimation	16 × 1.5
Normalized pitch	1

**Table 2:** Contrast administration parameters for both the CT/CEM and CEM-only groups.

Parameter	CT/CEM Group	CEM-Only Group
Temporal reference point	Contrast injection start defined as T0	Contrast injection start defined as T0
Contrast agent	Iopamidol 300 mg I/mL	Iopamidol 300 mg I/mL
Contrast dose	1.5 mL/kg	1.5 mL/kg
Total iodine load	450 mg I/kg	450 mg I/kg
Injection rate	2.5 mL/s	2.5 mL/s
Total injection duration	36–51 s	37–54 s
Saline flush	30 mL at 2.5 mL/s	30 mL at 2.5 mL/s
CT acquisition timing	90–120 s after T0	Not applicable
Patient transfer time (CT to CEM unit)	Mean 90 ± 20 s	Not applicable
Interval from T0 to first CEM exposure	Mean 4.20 ± 0.53 min	Mean 2.10 ± 0.43 min

**Table 3:** Imaging parameters for CEM examination.

Variable	Low-Energy	High-Energy
Filter type	Rh or Ag	Cu
Tube voltage (kVp)	26–31	45–49

**Table 4:** Distribution of biopsy results for the study cases. DCIS: ductal carcinoma in situ; IC-NOS: invasive cancer—not otherwise specified; IDC: invasive ductal cancer; ILC: invasive lobular cancer.

Histopathologic Type	CT/CEM Group, *n* (%)	CEM Group, *n* (%)	Total, *n* (%)
DCIS	0 (0%)	1 (2.9%)	1 (1.6%)
IC-NOS	1 (3.4%)	2 (5.9%)	3 (4.8%)
IDC	23 (79.3%)	24 (70.6%)	47 (74.6%)
IDC + ILC	1 (3.4%)	2 (5.9%)	3 (4.8%)
ILC	3 (10.3%)	4 (11.8%)	7 (11.1%)
Mucinous cancer	1 (3.4%)	1 (2.9%)	2 (3.2%)

**Table 5:** Evaluation of groups in terms of age and maximum diameter of the primary lesion.

	CT/CEM Group, Mean ± SD	CEM Group, Mean ± SD	*p*
Age	55.21 ± 11.69	55.67 ± 11.96	0.876
Lesion diameter	42.82 ± 23.03	29.15 ± 15.95	0.008 *

Student’s *t*-test. * *p* < 0.05.

**Table 6:** Comparison of lesion enhancement, background parenchymal enhancement (BPE), and lesion conspicuity between the groups (*p* > 0.05).

		CT/CEM	CEM	
		*n* (%)	*n* (%)	*p*
Lesion enhancement	Not present (0–1)	0 (0%)	2 (5.9%)	^1^ 0.495
	Present (2–3)	29 (100%)	32 (94.1%)	
Parenchymal enhancement	Not present (0–1)	18 (62.1%)	20 (58.8%)	^2^ 0.997
	Present (2–3)	11 (37.9%)	14 (41.2%)	
Lesion conspicuity	Not present (0–1)	4 (13.8%)	3 (8.8%)	^1^ 0.694
	Present (2–3)	25 (86.2%)	31 (91.2%)	

^1^ Fisher’s Exact test. ^2^ Continuity (Yates) correction.

**Table 7:** Evaluation of lesion enhancement and lesion conspicuity according to background parenchymal enhancement in the CT/CEM group.

		Parenchymal Enhancement		
CT/CEM Group		Not Present (0–1) *n* (%)	Present (2–3) *n* (%)	*p*
Lesion enhancement	Not present (0–1)	0 (0%)	0 (0%)	-
	Present (2–3)	18 (100%)	11 (100%)	
Lesion conspicuity	Not present (0–1)	0 (0%)	4 (36.4%)	0.014 *
	Present (2–3)	18 (100%)	7 (63.6%)	

Fisher’s Exact test. * *p* < 0.05.

**Table 8:** Evaluation of lesion enhancement and lesion conspicuity according to background parenchymal enhancement in the CEM-only group.

		Parenchymal Enhancement		
		Not Present (0–1)	Present (2–3)	
CEM Group		*n* (%)	*n* (%)	*p*
Lesion enhancement	Not present (0–1)	2 (10%)	0 (0%)	0.501
	Present (2–3)	18 (90%)	14 (100%)	
Lesion conspicuity	Not present (0–1)	0 (0%)	3 (21.4%)	0.061
	Present (2–3)	20 (100%)	11 (78.6%)	

Fisher’s Exact test.

**Table 9:** Assessment of additional lesions detected via CEM, degree of contrast enhancement and conspicuity of additional lesions, and lymph node involvement according to groups.

		CT/CEM Group	CEM Group	
		*n* (%)	*n* (%)	*p*
Additional lesion	Not present	22 (75.9%)	29 (85.3%)	^1^ 0.530
	Present	7 (24.1%)	5 (14.7%)	
Additional lesion enhancement	Not present (0–1)	0 (0%)	2 (40%)	^2^ 0.152
	Present (2–3)	7 (100%)	3 (60%)	
Additional lesion conspicuity	Not present (0–1)	0 (0%)	0 (0%)	-
	Present (2–3)	7 (100%)	5 (100%)	
Lymph node involvement		13 (44.8%)	15 (44.1%)	^1^ 1.000
		16 (55.2%)	19 (55.9%)	

^1^ Continuity (Yates) correction. ^2^ Fisher’s Exact test.

**Table 10:** Diagnostic performance of CEM for detecting lymph nodes and additional lesions, according to the biopsy results.

Group	Lesion Type	Sensitivity (%)	Specificity (%)	PPV (%)	NPV (%)	Accuracy (%)	Kappa	*p*-Value
Case Group	Additional lesion	85.7	95.5	85.7	95.5	93.1	0.313	0.089
Case Group	Lymph node	71.4	60.0	62.5	69.2	65.5		
Control Group	Additional lesion	80.0	96.6	80.0	96.6	94.1	0.467	0.006
Control Group	Lymph node	77.8	68.8	73.7	73.3	73.5		

PPV: Positive Predictive Value; NPV: Negative Predictive Value.

## Data Availability

The original contributions presented in this study are included in the article. Further inquiries can be directed to the corresponding author.

## Abbreviations

The following abbreviations are used in this manuscript:

CEM	Contrast-Enhanced Mammography
CT/CEM	Contrast-Enhanced Computed Tomography
BPE	Background Parenchymal Enhancement
IV	Intravenous
CC	Craniocaudal
MLO	Mediolateral Oblique

## References

[1] Benitez Fuentes J.D., Morgan E., de Luna Aguilar A., Mafra A., Shah R., Giusti F., Vignat J., Znaor A., Musetti C., Yip C.-H. (2024). Global Stage Distribution of Breast Cancer at Diagnosis: A Systematic Review and Meta-Analysis. JAMA Oncol..

[2] Poenaru M.O., Amza M., Toma C.V., Augustin F.E., Pacu I., Zampieri G., Ples L., Sima R.M., Diaconescu A.S. (2025). Multicentric and Multifocal Breast Tumors-Narrative Literature Review. Cancers.

[3] Lee-Felker S.A., Tekchandani L., Thomas M., Gupta E., Andrews-Tang D., Roth A., Sayre J., Rahbar G. (2017). Newly diagnosed breast cancer: Comparison of contrast-enhanced spectral mammography and breast MR imaging in the evaluation of extent of disease. Radiology.

[4] Chung W.-S., Tang Y.-C., Cheung Y.-C. (2024). Contrast-Enhanced Mammography: A Literature Review of Clinical Uses for Cancer Diagnosis and Surgical Oncology. Cancers.

[5] Açar Ç.R., Orguc S. (2024). Comparison of Performance in Diagnosis and Characterization of Breast Lesions: Contrast-Enhanced Mammography Versus Breast Magnetic Resonance Imaging. Clin. Breast Cancer.

[6] Nissan N., Sung J.S. (2025). Contrast-Enhanced Mammography: Advances, Challenges, and Case-Based Insights. Korean J. Radiol..

[7] Fallenberg E.M., Dromain C., Diekman F., Engelken F., Krohn M., Singh J.M., Ingold-Heppner B., Winzer K.J., Bick U., Renz D.M. (2014). Contrast-enhanced spectral mammography versus MRI: Initial results in the detection of breast cancer and assessment of tumour size. Eur. Radiol..

[8] Cozzi A., Magni V., Zanardo M., Schiaffino S., Sardanelli F. (2022). Contrast-enhanced Mammography: A Systematic Review and Meta-Analysis of Diagnostic Performance. Radiology.

[9] Tagliafico A.S., Bignotti B., Rossi F., Signori A., Sormani M.P., Valdora F., Calabrese M., Houssami N. (2016). Diagnostic performance of contrast-enhanced spectral mammography: Systematic review and meta-analysis. Breast.

[10] Neeter L.M.F.H., Robbe M.M.Q., van Nijnatten T.J.A., Jochelson M.S., Raat H.P.J., Wildberger J.E., Smidt M.L., Nelemans P.J., Lobbes M.B.I. (2023). Comparing the Diagnostic Performance of Contrast-Enhanced Mammography and Breast MRI: A Systematic Review and Meta-Analysis. J. Cancer.

[11] Gelardi F., Ragaini E.M., Sollini M., Bernardi D., Chiti A. (2022). Contrast-Enhanced Mammography versus Breast Magnetic Resonance Imaging: A Systematic Review and Meta-Analysis. Diagnostics.

[12] Jochelson M.S., Lobbes M.B.I. (2021). Contrast-enhanced Mammography: State of the Art. Radiology.

[13] Matthew F.C., Samantha S., Bradley D.W., Laurie L.F. (2024). State-of-the-art for contrast-enhanced mammography. Br. J. Radiol..

[14] Tsarouchi M., Hoxhaj A., Portaluri A., Sung J., Sechopoulos I., Pinker-Domenig K., Mann R.M. (2025). Breast cancer staging with contrast-enhanced imaging. The benefits and drawbacks of MRI, CEM, and dedicated breast CT. Eur. J. Radiol..

[15] Okada N., Tatsugami F., Sugai M., Okita I., Ito M., Ohtani S., Ichimura K., Urashima M., Awai K. (2019). The feasibility of contrast-enhanced spectral mammography immediately after contrast-enhanced CT. Radiol. Phys. Technol..

[16] Zhu X., Huang J.M., Zhang K., Xia L.J., Feng L., Yang P., Zhang M.Y., Xiao W., Lin H.X., Yu Y.H. (2018). Diagnostic Value of Contrast-Enhanced Spectral Mammography for Screening Breast Cancer: Systematic Review and Meta-analysis. Clin. Breast Cancer.

[17] Bechyna S., Santonocito A., Pötsch N., Clauser P., Helbich T.H., Baltzer P.A.T. (2025). Impact of Background Parenchymal Enhancement (BPE) on diagnostic performance of Contrast-Enhanced Mammography (CEM) for breast cancer diagnosis. Eur. J. Radiol..

[18] Allajbeu I., Nanaa M., Manavaki R., Papalouka V., Bene I., Payne N., Giannotti E., van Nijnatten T., Kilburn-Toppin F., Healy N. (2025). Improving the diagnostic performance of contrast-enhanced mammography through lesion conspicuity and enhancement quantification. Eur. Radiol..

[19] Nissan N., Sevilimedu V., Gluskin J., Arita Y., Keating D.M., D’Alessio D., Fruchtman-Brot H., Ochoa-Albiztegui R.E., Sung J.S., Jochelson M.S. (2025). Hormonal Regulation of Background Parenchymal Enhancement at Contrast-enhanced Mammography. Radiology.

[20] Wang S., Sun Y., You C., Jiang T., Yang M., Shen X., Qian M., Duan S., Lynn H.S., Li R. (2023). Association of Clinical Factors and Degree of Early Background Parenchymal Enhancement on Contrast-Enhanced Mammography. AJR Am. J. Roentgenol..

[21] Meucci R., Pistolese C.A., Perretta T., Vanni G., Beninati E., Tosto F.D.I., Serio M.L., Caliandro A., Materazzo M., Pellicciaro M. (2022). Background Parenchymal Enhancement in Contrast-enhanced Spectral Mammography: A Retrospective Analysis and a Pictorial Review of Clinical Cases. In Vivo.

[22] Neeter L.M.F.H., Raat H.P.J.F., Alcantara R., Robbe Q., Smidt M.L., Wildberger J.E., Lobbes M.B.I. (2021). Contrast-enhanced mammography: What the radiologist needs to know. BJR Open.

[23] Zanardo M., Cozzi A., Trimboli R.M., Labaj O., Monti C.B., Schiaffino S., Carbonaro L.A., Sardanelli F. (2019). Technique, protocols and adverse reactions for contrast-enhanced spectral mammography (CESM): A systematic review. Insights Imaging.

[24] Jochelson M.S., Dershaw D.D., Sung J.S., Heerdt A.S., Thornton C., Moskowitz C.S., Ferrara J., Morris E.A. (2013). Bilateral contrast-enhanced dual-energy digital mammography: Feasibility and comparison with conventional digital mammography and MR imaging in women with known breast carcinoma. Radiology.

[25] Helal M.H., Mansour S.M., Salaleldin L.A., Alkalaawy B.M., Salem D.S., Mokhtar N.M. (2018). The impact of contrast-enhanced spectral mammogram (CESM) and three-dimensional breast ultrasound (3DUS) on the characterization of the disease extend in cancer patients. Br. J. Radiol..

[26] Ancona A., Telegrafo M., Fella R.R., Iamele D., Cantore S., Moschetta M. (2024). CEM immediately after contrast-enhanced CT: A one-step staging of breast cancer. Eur. Radiol. Exp..

[27] Wong C.Y.Y., Lee S.Y.S., Mahmood R.D. (2024). Contrast-enhanced spectral mammography. Singap. Med. J..

